# [Expression of Concern] Metformin inhibits growth and sensitizes osteosarcoma cell lines to cisplatin through cell cycle modulation

**DOI:** 10.3892/or.2026.9116

**Published:** 2026-04-08

**Authors:** Irene Quattrini, Amalia Conti, Laura Pazzaglia, Chiara Novello, Stefano Ferrari, Piero Picci, Maria Serena Benassi

Oncol Rep 31: 370–375, 2014; DOI: 10.3892/or.2013.2862

Following the publication of the above paper, it was drawn to the Editor's attention by a concerned reader that, regarding the flow cytometric plots shown in Fig. 3 on p. 372, the C/U2OS and 48 h/U2OS, C/143B and 48 h/143B, and C/MG63 and 72 h/143B data panels, respectively, were strikingly similar, suggesting that this figure has been assembled incorrectly, and that these data were derived from a smaller number of original sources.

The authors have been contacted by the Editorial Office to offer an explanation for the apparent duplication of these data panels within this figure, and we are waiting their response. Owing to the fact that the Editorial Office has been made aware of potential issues surrounding the scientific integrity of this paper, we are issuing an Expression of Concern to notify readers of this potential problem while the Editorial Office continues to investigate this matter further.

